# Genetic Determinants and Biofilm Properties Useful in Estimation of UTI Pathogenicity of the *Escherichia coli* Strains Isolated from Free-Living Birds

**DOI:** 10.3390/antibiotics14010032

**Published:** 2025-01-03

**Authors:** Bartosz Rybak, Tomasz Jarzembowski, Agnieszka Daca, Beata Krawczyk, Lidia Piechowicz

**Affiliations:** 1 Department of Environmental Toxicology, Faculty of Health Sciences, Institute of Maritime and Tropical Medicine, Medical University of Gdańsk, Dębowa 23 A, 80-204 Gdańsk, Poland; 2 Department of Medical Microbiology, Faculty of Medicine, Medical University of Gdańsk, Dębowa 25, 80-204 Gdańsk, Poland; tjarzembowski@gumed.edu.pl (T.J.); lidia.piechowicz@gumed.edu.pl (L.P.); 3 Department of Physiopathology, Medical University of Gdańsk, Dębinki 7, 80-211 Gdańsk, Poland; agnieszka.daca@gumed.edu.pl; 4 Department of Biotechnology and Microbiology, Faculty of Chemistry, Gdańsk University of Technology, G. Narutowicza 11/12, 80-233 Gdańsk, Poland; beakrawc@pg.edu.pl

**Keywords:** UPEC, environmental strains, Clermont phylogroups, biofilm formation, monocytes activation, *E. coli* pathogenicity

## Abstract

**Background/Objectives**: According to the One Health concept, wild birds can be indicators of ecosystem pollution and disease incidence. *Escherichia coli* strains are widespread worldwide, but there are still few reports on the association of human infections with a potential reservoir of highly pathogenic human strains in wild birds. Fecal *E. coli* with uropathogenic potential (UPEC) can be transmitted between birds and humans and may be a risk factor for urinary tract infections (UTIs). **Results**: The results showed that above 50% of the isolates were grouped as highly pathogenic, according to Clermont phylogroup classification. Such strains were found to be stronger biofilm producers, with a higher adherence of monocytes than low pathogenic. However, the highest cytotoxicity was observed for strains described as aquatic environmental. Convergence of the results of the analysis of monocyte activation by *E. coli* strains and the ability to form biofilm by individual phylogroups of the strains tested was demonstrated. Genetic determinants of the uropathogenicity of *E. coli* (UPEC) correlate with the evidence of strain pathogenicity during monocyte activation in in vitro assays. **Methods**: In this study, we assessed the virulence potential of environmental strains isolated from wild waterfowl using genetic analysis (Clermont phylogroup classification) and phenotypic methods, including analysis of the human monocyte response to biofilm formation. The estimation of the ability to form biofilms was tested using crystal violet, and the pathogenic potential of strains by monocyte activation assay including changes in morphology, adhesion and cytotoxicity. **Conclusions**: In conclusion, the virulence of *E. coli* strains isolated from free-living birds is significant, and they can be considered environmental reservoirs of pathogenic strains. According to our observations, they can be responsible for the dissemination of uropathogenic strains among humans.

## 1. Introduction

*Escherichia coli* is a common bacterium of fecal origin found in various etiological niches in the external environment. It can be found in salt and fresh water, lakes, rivers, ponds and agricultural soils, and it was isolated both from urban areas and forests [1]. *E. coli* is also considered a highly pathogenic bacterium. The prevalence of *E. coli* strains and their high biodiversity, including the diversity of genetic determinants of virulence, forces them to be considered as etiological factors of urinary tract infections (UTIs) in humans. The high prevalence of strains in various ecological ecosystems is associated with the possibility of migration between different areas due to colonization in the digestive tracts of wild animals, farm animals and humans. Fecal contamination is the main source of *E. coli* strains in the environment. Fecal strains originated from the digestive tracts of humans (dispersed around the environment by residues from the treatment process in sewage treatment plants), livestock and wild animals. As to the etiology of UTIs, the source of *E. coli* strains is mainly the endogenous microbiota of patients, both drug-sensitive and drug-resistant [2]. Epidemiological analyses of UTI factors should include environmental strains of *E. coli* with possible infectious potential. Genetic relationships between avian pathogenic *E. coli* (APEC) and uropathogenic *E. coli* (UPEC) isolates are increasingly reported [3,4,5].

Virulence factors (Vfs) of *E. coli* are important in severe UTIs. Some of the virulence genes of UPEC strains are siderophores, e.g., enterobactin; salmochelin; yersiniabactin and aerobactin; fimbriae, e.g., type 1, P, S, F1C; Dr family of adhesins afimbrial adhesin 1 Afa1; and toxins, e.g., cytotoxic necrotizing factor 1 (CNF) and α-hemolysin (HlyA) [6]. Biofilm forming is a very important ability of UPEC because it allows the survival of bacterial cells in the urinary tract and the persistence of bacterial infection [7]. In addition to fimbriae, the role of Ag43 and IbeA proteins has been described in the first stages of attachment and aggregation [8]. Some virulence genes are located on the pathogenicity islands (PAIs) or plasmids and can be transferred to other strains by horizontal gene transfer, typical of *E. coli* strains [9]. Due to the different degrees of variability of Vfs, a division of *E. coli* strains into phylogenetic groups was established, namely, commensal and pathogenic. According to Clermont et al., *E. coli* strains can be categorized into seven *sensu stricto* phylogenetic groups:—A, B1, B2, C, D, E, F—while the eighth group is called a cryptic *Escherichia* clade I [10]. Commensal *E. coli* colonize the gastrointestinal tract mucosa and most often represent groups A or B1. Some strains of phylogroup B1 were reported to persist in water and soil [11]. The pathogenic strains responsible for intestinal infections can also be represented by A, B1 and group D (for example DEC—Diarrheagenic *Escherichia coli*), while most extraintestinal pathogenic *E. coli* (ExPEC) strains including UPEC, septicemia-associated *E. coli* (SEPEC) and neonatal meningitis-associated *E. coli* (NEMEC) belong to phylogenetic group B2, followed by group D [12]. Categorization of *E. coli* strains into phylogenetic groups can be a useful method of strain differentiation as pathogenic and commensal [13,14]. APEC strains are a separate group belonging to the potential foodborne zoonotic pathogens. They cause colibacillosis in birds [15] but may be genetically similar to the human pathotypes listed above. APEC is also a pathogen that is important from the point of view of public health, due to its transmission from poultry to human beings [16].

Wild birds can be colonized by bacteria, which are often resistant to antibiotics and have various virulence profiles [14,17,18]. Birds migrating over long distances and locally in search of food are an important vector for the transmission of drug-resistant and pathogenic bacterial strains for human infections [19,20].

The development of *Homo sapiens* societies and their spreading around the world is indicative of our dominance as a species. The impact of the byproducts of our activity—namely, pollutants entering the environment—is well-known and mainly negative. It is established knowledge as the indicators of both biological and chemical pollution, as well as of the changes in the habitats, such as the increased diseases incidence in the wild birds can be treated. It is also well known that farm animals can be a source of infectious agents for humans. The implication of wild birds in the same phenomenon is not as well proved.

Nowadays, the assessment of the amount of various anthropogenic pollutants entering the environment is of key importance. Wild birds can be indicators of ecosystem health, reflecting changes in habitats, increased disease incidence, and exposure and effects of biological and chemical pollution. The role of birds in the transmission of infectious diseases is well established, and their ability to harbor a wide range of pathogens (*Campylobacter* spp., *Yersinia* spp., *C. difficile*, enteropathogenic *E. coli*) has been documented by numerous studies. Globally, the movement of free-living birds, coupled with environmental stressors and human interaction, amplifies the potential for the transmission of bacterial pathogens to both avian and human populations [21]. The relationship between humans and farm animals has already been proven many times, but there are still only a few reports on the association of infections in humans with a potential source among wild birds.

Traditional microbiological diagnosis is based on the bacteriological culture of a urine sample from a patient with symptoms or a suspected UTI. A positive result is considered to be the cultivation of a uropathogen at an appropriate titer. However, to our knowledge, it is not possible to distinguish between asymptomatic bacteriuria and a colonization prior infection with a low-virulence commensal strain of *E. coli*. In routine bacteriological diagnostics, the determination of virulence genes, phylogroups or the ability to form a biofilm on the bladder wall is ineffectual and takes too long to be an effective clinical procedure. As the monocytes/macrophages are always one of the first reacting to the presence of the foreign element in the infected area, apart from neutrophils, and the monocytes’ plasticity and quickness in the developing immune reaction is well known, we decided to use them as the possible indicators of the pathogenicity of analyzed bacterial strains. Our earlier studies proved already that the determination of parameters describing the interaction between monocytes and bacteria, such as the changes in monocytes’ size and their adhesive properties, can in fact estimate the virulence of bacteria [22]. The result of the interaction between monocytes and bacteria isolated from urine samples of patients with suspected urinary tract infections can reflect on the virulence of the bacteria and help in the planning of the treatment of UTI [22]. The monocytes’ changes in size and granularity allow them to quickly phagocytose the offending agent, and the strength of the observed response depends on the pathogenicity of the encountered bacteria. As the first step to phagocytose a foreign element is to adhere to it, that ability in itself can be considered as a useful tool to assess the pathogenicity of the analyzed strain, bypassing the time-consuming techniques [23]. 

The aim of this study was to evaluate the virulence potential of environmental *E. coli* strains isolated from wild birds as potential etiological factors in urinary tract infections. The virulence potential was assessed by biofilm formation ability, Clermont phylogroups analysis and analysis of the monocytes’ response to the biofilm formed.

## 2. Results

Twenty-three strains of *E. coli* isolated from swabs from the cloaca of waterbirds collected in the Pomeranian region, Poland, were subjected to the study [14]. Table S1 shows the studied species of birds, the origin of the strains, a pattern of susceptibility and the pathogenicity. Bacterial strains used in this study were deposited in the Collection of Plasmids and Microorganisms, KPD, University of Gdansk, Gdansk, Poland.

The presence of ESBL beta-lactamases was confirmed by the double-disk diffusion phenotypic method in all strains.

**Figure 1:**
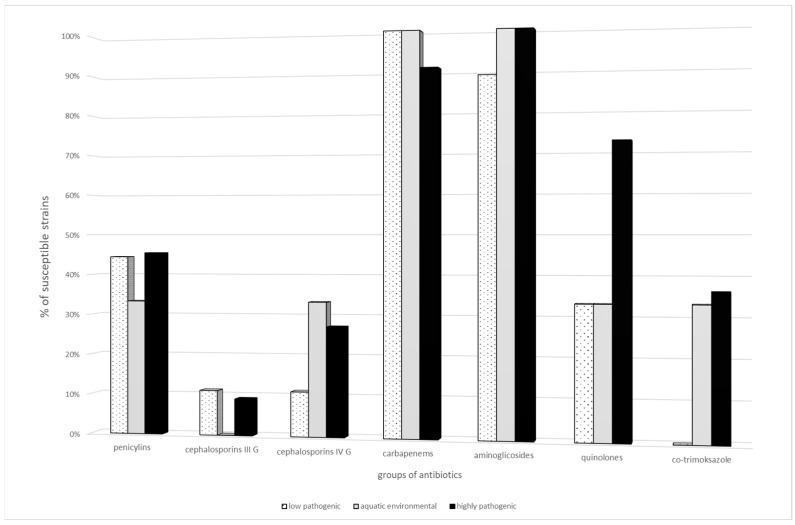
Drug susceptibility phenotypes of the tested *E. coli* strains (phylogroups by Clearmont—low pathogenic l: A; aquatic environmental: B1; highly pathogenic: B2, D).

Among the analyzed antibiotics, the tested strains showed the highest resistance to cephalosporins, but cefepime showed the highest activity against the strains tested. No strains resistant to carbapenems have been reported. High levels of resistance were demonstrated among penicillins and their combinations with beta-lactamase inhibitors, with the exception of piperacillin with tazobactam, to which less than 50% of the strains were susceptible. High levels of susceptibility to aminoglycoside drugs, fluoroquinolones and co-trimoxazole have been reported. A detailed analysis of the susceptible strains is shown in Figure 1.

Avian cephalosporin-resistant (CF-R) *E. coli* belong to diverse phylogenetic groups. Nine strains belonged to group A (low pathogenic), three strains to group B1 (aquatic environmental), five strains to group B2 (highly pathogenic) and seven strains to group D (highly pathogenic) [14]. Phylogroups in relation to pathogenicity are shown in Table 1.

**Figure 2:**
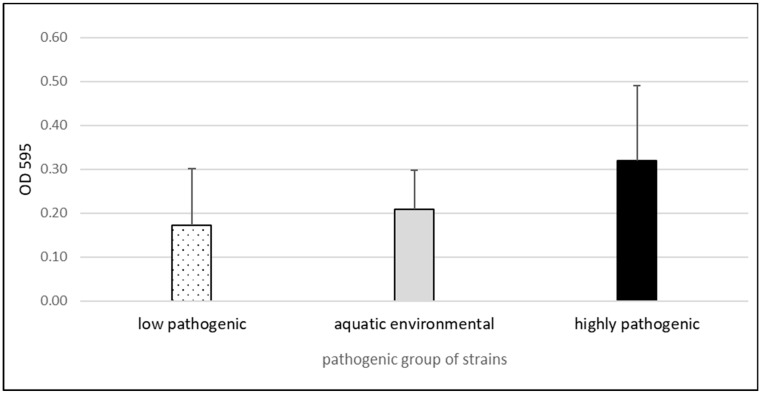
Results of the determination of the ability to form a biofilm with the use of crystal violet at the temperature of 37 °C (phylogroups by Clearmont—low pathogenic: A; aquatic environmental: B1; highly pathogenic: B2, D).

The analysis of the ability to form biofilm with the use of crystal violet showed a high degree of diversity among the analyzed strains. The comparison of the ability to form biofilm between phylogroups showed a greater ability in highly pathogenic strains and the lowest in low pathogenic strains. Detailed results are presented in Figure 2.

**Figure 3:**
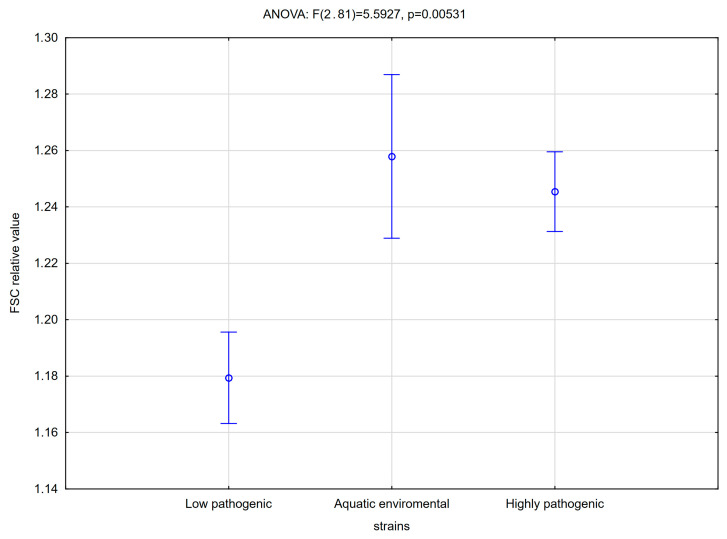
Changes in the relative size of monocytes after the exposition to biofilm formed by tested strains. Vertical bars represent standard error (phylogroups by Clearmont—low pathogenic: A; aquatic environmental: B1; highly pathogenic: B2, D).

The difference in the biofilm properties, in particular, phylogenetic groups of strains, include both the amount of biofilm (Figure 2) and its ability to activate human monocytes (Figure 3). As reported previously [23], isolates from significant bacteriuria formed biofilm, which stimulated the increase of the size of monocytes (FSC, forward scatter). Here, the same result was observed for highly pathogenic strains and aquatic environmental strains (Figure 3).

Effects of the analyzed strains on monocytes were determined based on the assessment of the level of adhesion, change in monocyte size and cytotoxicity. The highest median values for adhesion (0.85, Mann–Whitney U *p* < 0.05 Z = −2.66), changes in monocyte size (1.26, Mann–Whitney U *p* > 0.05 and Z = −3.33) and cytotoxicity (6.28, Mann–Whitney U *p* < 0.05 Z = −1.99) are reported among strains classified into phylogroups of aquatic environmental strains, while the lowest values for adhesion (0.66, Mann–Whitney U *p* < 0.05 Z = −2.66) are for strains classified as highly pathogenic phylogroups, and the lowest cytotoxicity (1.18, Mann–Whitney U *p* < 0.05 Z = −3.33) is for low pathogenic strains.

**Figure 4:**
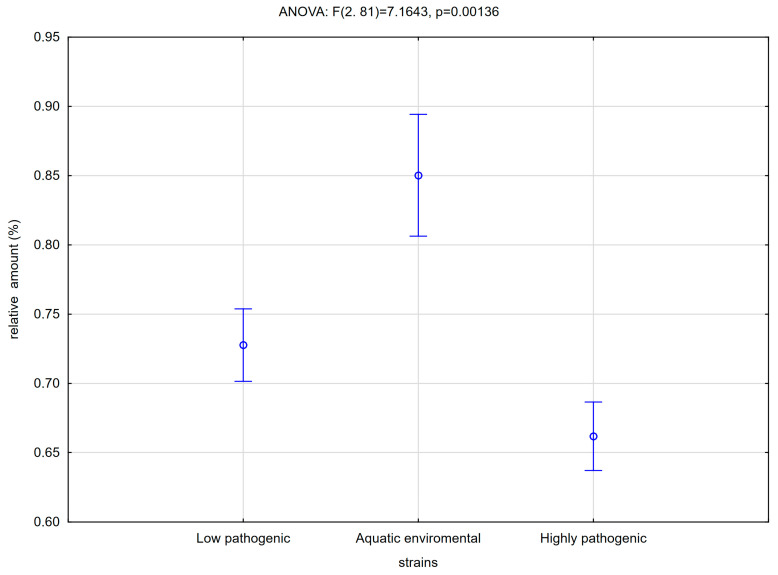
Comparison of adherence of monocytes to bacterial biofilm formed by strains recognized as low pathogenic, environmental or highly pathogenic by phylogenetic analysis (ANOVA). Vertical bars represent standard error (phylogroups by Clearmont—low pathogenic: A; aquatic environmental: B1; highly pathogenic: B2, D).

**Figure 5:**
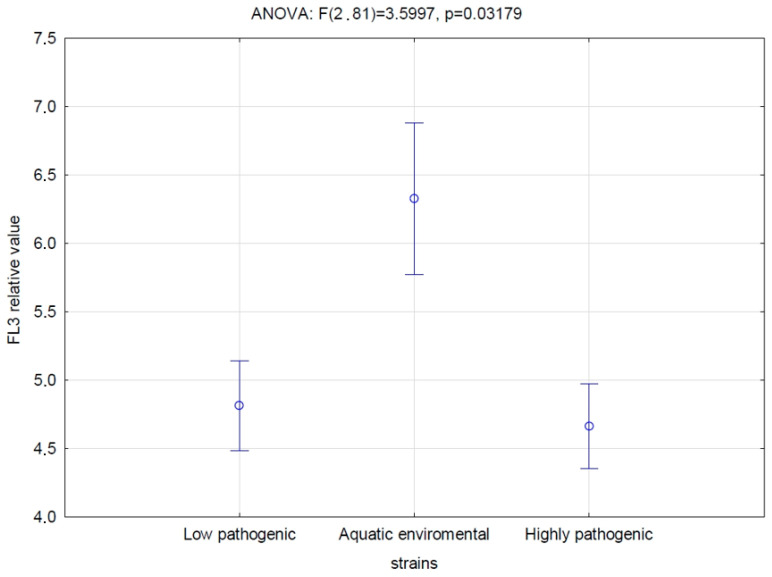
Cytotoxicity of bacterial biofilm formed by the strains recognized as low pathogenic, environmental or highly pathogenic by phylogenetic analysis. The value was estimated by monocyte cell membrane permeability after the exposition to the biofilm and measured as an increase of red fluorescence after PI staining. Vertical bars represent standard error (phylogroups by Clearmont: low pathogenic: A; aquatic environmental: B1; highly pathogenic: B2, D).

Likely, the changes in biofilm structure also result in the adherence of monocytes to such structure. As a result, the recovery of monocytes from the suspension was lower for the phylogenetic group of highly pathogenic strains (Figure 4) and isolates from patients with a UTI infection [22]. What is surprising, though, is that the recovery of monocytes from biofilm formed by the environmental phylogenetic group of strains was even higher than for the human low pathogenic strain (Figure 4). Additionally, this group of strains used to have the highest cytotoxicity, while there was no difference between highly pathogenic and low pathogenic phylogenetic groups of strains (Figure 5).

Multifactorial analysis shows that aquatic strains have the most intense interactions with monocytes, and they were significant producers of biofilm in the crystal violet test. Highly pathogenic strains showed more intensive production of biofilm but recovery from biofilm was not high.

## 3. Discussion

One of the most common outpatient uses of antibiotics is to treat urinary tract infections, usually caused by *E. coli*. Although antibiotic-resistant pathogens are an increasing challenge to the care of hospital inpatients, more than half of antibiotic use in human healthcare occurs in an outpatient setting [24]. The main etiological factors of UTI are *E. coli* strains of endogenous origin. Environmental strains, in particular, from aquatic environments, including those transmitted by wild birds, should also be considered as etiologic factors of UTI in humans, especially in patients with acquired or congenital compromised immunity [25,26,27].

As expected, strains typical of the aquatic environment were found in our previous study [14] and other studies [24,28,29,30], but contrary to our expectations, a large percentage of strains belonged to the phylogenetic group with highly pathogenic potential [14,30].

In this study, 23 strains of CF-R *E. coli* were analyzed from another larger project to analyze the occurrence of CF-R strains, in which 241 wild birds were studied, from which 23% of strains producing extended-spectrum beta-lactamases (ESBL) were isolated. In our research, CF-R avian *E. coli* strains were assigned to the phylogroups by Clearmont—low pathogenic: A; commensal for animals or aquatic environmental: B1; high pathogenic: B2, D [10]. Strains from group B2 (assumed to be highly pathogenic) were characterized by the highest biofilm biomass, as opposed to low pathogenic strains from phylogroup A. The above results are consistent with the results of monocytes’ recovery after exposition (estimation after recovery) to biofilm. In contrast to adhesion, the results of changes in monocytes’ size and cytotoxicity for individual phylogroups were higher for highly pathogenic strains belonging to groups D and B2. If we accept, in accordance with the majority of authors, the involvement of biofilm in pathogenicity [31,32], this proves the variability of both the potential and actual virulence of these strains. It is worth noticing that aquatic strains had a surprisingly high cytotoxicity. Considering them as a likely threat to the public, it is especially worth additional investigation.

The pathogenic potential of these strains was also confirmed by UriMAB (Urine bacteriology diagnostics based on monocyte activation by biofilm), the technique that showed increased adhesion; however, environmental strains showed the greatest cytotoxicity in this method. To our knowledge, such results have not yet been published.

The analysis of the drug resistance of the strains indicates possible therapeutic difficulties in UTI infections caused by these strains. Resistance to third-generation cephalosporins is a significant risk of treatment failure in empiric therapy of complicated UTIs in patients, involving hospitalization. In addition, variable resistance to other groups of antibiotics is a threat of treatment failure. Plasmids carrying ESBL resistance genes may also carry genes for resistance to other drug classes in parallel, including fluoroquinolones, sulfonamides or aminoglycosides [33,34,35,36,37].

This study was designed to evaluate the pathogenic potential of *E. coli* strains isolated from free-living birds to cause UTI infections in humans. Infections could occur by bathing on the beaches where the tested birds were caught, but also by not maintaining proper hand hygiene when moving through different environments. Antibiotic-resistant bacteria are widespread in the aquatic environment, as shown by numerous studies, but the current sanitary regulations in the EU do not monitor this phenomenon on a routine basis. An assessment of the potential for exposure to organisms of public health concern in naturally occurring bathing waters in Europe indicates a potential risk from these strains to the health and life of the population [38].

The occurrence of CF-R strains in the natural environment has been confirmed in numerous studies, in particular in various surface water reservoirs (lakes, rivers of the sea) [39,40,41,42]. Is the aquatic environment conducive to the acquisition of human strains/colonization, in particular, of the gastrointestinal tract, by drug-resistant strains, including CF-R? Water reservoirs in which a person rests/relaxes may be a source of initially harmless colonization of people/society by drug-resistant strains. These strains, while carrying the mobile determinants of drug resistance, do not have to be highly pathogenic, but they can transmit resistance. If we accept, in accordance with the majority of authors, the involvement of biofilm in pathogenicity mechanisms of the resistance genes transfer into the strains of the natural intestinal flora through transduction or transformation, the issue becomes hard to ignore. All of this concerns an increased risk of therapeutic failure of infections caused by *E. coli* in immunocompromised patients, but not only.

Among wild birds, drug-resistant strains, including CF-R strains, were found [39,43]. The isolated CF-R strains from birds belonged to numerous species, including *E. coli*, which is considered to be the major etiological agent of UTIs. The spread of CF-R strains in the aquatic, urban or human environment by wild birds poses a risk of infections with treatment difficulties for patients with acquired or inherited compromised immunity. Wild-living birds can also be vectors that carry strains of drug-resistant CF-R bacteria, e.g., *E. coli,* from various habitats, leading to the contamination of water reservoirs used for reaction baths with uropathogenic strains. Susceptible persons may become infected or colonized with such strains, thereby increasing their risk of UTI with a CF-R strain, insensitive to cephalosporins, used as an empirical drug for the treatment of UTIs in a patient with community-acquired urosepsis [44]. Due to the small number of studies, it is difficult to confirm the direct relationship that zoonotic transmission of *E. coli* from birds to humans might occur. By comparing the facts of the proved existence of wild birds, such as ducks and seagulls, colonized in the digestive tract with CF-R strains with the presence of such CF-R strains in reservoirs where they live, one can draw a conclusion about the relationship between wild birds and infections in people resting by these reservoirs, having contact with water and sand and forgetting about the principles of hygiene. Our previous studies have shown a wide distribution of CF-R strains in reservoirs and watercourses, correlating with the occurrence of CF-R-positive birds in them [44].

There are not many studies available on the pathogenicity of *E. coli* CF-R strains isolated from wild birds or their potential threat as etiological agents of UTI. This paper highlights the need to consider environmental strains in the analysis of etiological factors of UTI in humans, especially in immunocompromised individuals.

## 4. Materials and Methods

### 4.1. Strains

Twenty-three environmental strains of CF-R *E. coli* from previous studies were selected for analysis. The strains were derived from the collection of the Department of Environmental Toxicology of the Medical University of Gdańsk and were previously deposited in the Collection of Plasmids and Microorganisms of the University of Gdańsk and were isolated from a large study analyzing the occurrence and characteristics of cephalosporin-resistant strains in the gastrointestinal tract of wild waterfowl in northern Poland [14] Drug susceptibility testing was conducted according to the European Committee for Antimicrobial Susceptibility Testing (EUCAST) v.10.0 (2020) guidelines [45], and phylogenetic analysis was performed by the method developed by Clermont et al. [10], by multiplex PCR of the genes *chu*A, *yja*A and the DNA fragment TSPE4.C2., in accordance with previous descriptions [10,14,46].

### 4.2. Biofilm Formation Assays

The biofilm formation assay was performed as in Nykyri et al. (2013), with some modifications. A total of 1 mL of dilute overnight bacterial culture to OD600 = 0.1 was inoculated into 24-well Nunclon Delta Surface (Thermo Fisher Scientific, Waltham Massachusetts, USA) plates and kept for 48 h at 37 °C without agitation [47]. After the incubation period, OD600 of bacterial cultures was measured, and the planktonic bacterial cultures were removed from the wells, and the formed biofilm remained. Then, 1 mL of 1% crystal violet solution was added into each well and left for 20 min without agitation. After incubation, the wells were washed 3 times with 900 μL of distilled water. Then, 900 μL of acetic acid (33%, Merck, Burlington, MA, USA) was added into each well, and OD_595_ of each well was measured. For calculations, the OD_595_ value of the negative control was subtracted from the values obtained for the strains. Negative values obtained after subtraction were set to 0. The experiment was performed three times with two replicates N [14].

### 4.3. Estimation of Pathogenic Potential of Strains by Monocyte Activation Assay

The studied strains were cultured at 37 °C in a container consisting of two polystyrene coupons for adhesion of bacteria and biofilm formation. To increase adherence of bacteria, one of the coupons was additionally coated with poly-L-lysine. The incubation lasted for 72 h in the BHI medium. After 24 h, half of the volume of the medium was replaced with the fresh one.

The preparation of the monocytes’ cell line (ATCC, TIB-202) for the activation assay was described earlier [22]. Shortly, the THP-1 cell line was cultured in RPMI-1640 medium supplemented with 2 mM L-glutamine, 100 U/mL penicillin, 100 μg/mL streptomycin and 10% (*vol*/*vol*) heat-inactivated fetal bovine serum (FBS) (all from Merck). For the activation assay, the monocytes were counted, and their vitality was checked using 0.4% trypan blue (Merck). After washing with sterile PBS (phosphate buffer saline) to eliminate the antibiotics, they were suspended in 0.9% NaCl to the final concentration of 0.125 × 10^6^/mL. Wells with biofilm were washed with 0.9% NaCl; a 2 mL suspension of monocytes was then added and incubated at 37 °C for 60 min. Sterile wells were used as a reference for adhesion and the activation of monocytes. After the incubation, the monocytes were stained with propidium iodide (PI). Subsequently, monocytes’ amount (counted per 60 s), size (described by the FSC—forward scatter—parameter) and cytotoxicity (registered by the FL3 channel in the flow cytometer, reflecting PI fluorescence) were estimated by the BD FACS Verse flow cytometer (Becton-Dickinson, Franklin Lakes, NJ, USA). The conditions of the monocytes’ exposition to bacterial biofilm including the assay of reproducibility has been evaluated previously [22].

### 4.4. Statistical Analysis

The ability to form biofilm by 3 phylogroups was analyzed. The differences within isolates were tested by an analysis of variance (ANOVA), while the differences between particular groups were confirmed by the Mann–Whitney U test by StatSoft software (Statistica 10, TIBCO Software, Santa Clara, CA, USA). The differences in results obtained by flow cytometry, namely, changes in the size of monocytes (FSC), their ability to adhere to the biofilm surface and the cytotoxicity of the analyzed strains, were analyzed by using the Mann–Whitney U test.

## 5. Conclusions

The coincidence of genetic properties, biofilm formation and activation of human monocytes could lead us to the conclusion of unique properties of UTI pathogens. As a result, it should be possible to distinguish between virulent strains likely to cause UTI and low pathogenic ones. However, in contrast to expectation, strains categorized as aquatic environments showed the highest cytotoxicity to human monocytes. So, further studies are needed to evaluate the interaction of strains of animal origin and the human immune system’s epithelial cells of the urinary tract, in particular.

## Figures and Tables

**Table 1:** Results of the effects of the analyzed strains on monocytes (median) (phylogroups by Clearmont—low pathogenic: A; aquatic environmental: B1; highly pathogenic: B2, D). FSC—forward scatter, FL3—the median value of propidium iodide fluorescence; Mann–Whitney U test.

Strain Phylogroup	Low Pathogenic	Aquatic Environmental	Highly Pathogenic	*p* < 0.05
Adhesion/monocyte recovery from biofilm [relative amount (%)]	0.73 (0.36–0.83)	0.85 (0.25–0.9) high	0.66 (0.23–0.69) low	Z = −2.66
Changes in size of monocyte [FSC relative value]	1.18 (1.06–1.37) low	1.26 (1.03–1.32)	1.24 (1.15–1.34)	Z = −3.33
Cytotoxicity [FL3 relative value]	4.81 (1.72–6.7)	6.28 (2.24–6.59) high	4.66 (2.75–5.55)	Z = −1.99

## Data Availability

The datasets generated and/or analyzed during the current study are available from the corresponding author on reasonable request.

## Supplementary Materials

The following supporting information can be downloaded at: https://www.mdpi.com/article/10.3390/antibiotics14010032/s1, Table S1: List of tested *E. coli* strains, their origin pathogenicity and pattern of susceptibility.(14).
