# Supplementary material for: Genetic Determinants and Biofilm Properties Useful in Estimation of UTI Pathogenicity of the Escherichia coli Strains Isolated from Free-Living Birds
Source: Antibiotics (Basel). 2025 Jan 3;14(1):32. doi: 10.3390/antibiotics14010032 (PMC11762073; doi:10.3390/antibiotics14010032)
Supplement: Supplementary file 1 [file antibiotics-14-00032-s001.zip › antibiotics-3349597-supplementary.pdf]

antibiotics

MDPI

Genetic determinants and biofilm properties useful in estimation of UTI pathogenicity of the *Escherichia coli* strains isolated from free-living birds.

Bartosz Rybak [1], Tomasz Jarzembowski [2], Agnieszka Daca [3], Beata Krawczyk [4] and Lidia Piechowicz [2]

Supplementary materials

Table S1. List of tested *E. coli* strains, their origin pathogenicity and pattern of susceptibility.(14).

KPD strain	No. bird rings	Bird species	Phylogroup	Pathogenicity	Pattern of susceptibility
KPD 932-BA	SN20132	*Anas platyrhynchos*	A	low pathogenic	TZP, MEM, IPM, ETP, AMK, CN, NET
KPD 929-BA	SN20593	*Anas platyrhynchos*	A	low pathogenic	TZP, MEM, IPM, ETP, AMK, CN, NET, TGC
KPD 942-BA	FS13072	*Chroicocephalus ridibundus*	A	low pathogenic	MEM, IPM, ETP, AMK, CN, NET
KPD 790-BA	SN25556 d	*Fulica atra*	A	low pathogenic	MEM, IPM, ETP, AMK, CN, NET
KPD 782-BA	SN25556 c	*Fulica atra*	A	low pathogenic	MEM, IPM, ETP, AMK, CN, NET, CIP, TGC
KPD 949-BA	SN26875A	*Fulica atra*	A	low pathogenic	TZP, FEP, CAZ, MEM, IPM, ETP, AMK, CIP, TGC
KPD 958-BA	Sn20598	*Anas platyrhynchos*	A	low pathogenic	MEM, IPM, ETP, AMK, CN, NET
KPD 935 BA	SN20593_2	*Anas platyrhynchos*	A	low pathogenic	TZP, MEM, IPM, ETP, AMK, CN, NET, CIP, TGC
KPD 926-BA	FS17247	*Larus canus*	B1	aquatic environmental	MEM, IPM, ETP, TGC
KPD 945-BA	SN26873B	*Fulica atra*	B1	aquatic environmental	MEM, IPM, ETP, AMK, CN, NET, CIP, SXT, TGC
KPD 960-BA	Sn20597	*Anas platyrhynchos*	B1	aquatic environmental	FEP, MEM, IPM, ETP, AMK, CN, NET, SXT
KPD 969BA	SN25556 b+	*Fulica atra*	B2	highly pathogenic	FEP, MEM, IPM, ETP, CN, NET, TGC
KPD 968BA	SN25556 b-	*Fulica atra*	B2	highly pathogenic	FEP, MEM, IPM, ETP, CN, NET, CIP, SXT, TGC
KPD 819-BA	DN20055	*Larus canus*	B2	highly pathogenic	FEP, MEM, IPM, ETP, AMK, CN, NET, TGC
KPD 943-BA	SN26872A	*Fulica atra*	B2	highly pathogenic	FEP, MEM, IPM, ETP, AMK, CN, NET, CIP, SXT, TGC
KPD 970 BA	SN25566	*Fulica atra*	B2	highly pathogenic	FEP, MEM, IPM, ETP, AMK, CN, NET, CIP, SXT, TGC
KPD 922-BA	SN20006	*Anas platyrhynchos*	A	low pathogenic	MEM, IPM, ETP
KPD 934-BA	DN20068	*Larus argentatus*	D	highly pathogenic	MEM, IPM, ETP, AMK, CN, NET, CIP
KPD 923-BA	DN20070	*Larus argentatus*	D	highly pathogenic	TZP, FEP, MEM, IPM, AMK, CIP, TGC
KPD 928-BA	SN20595	*Anas platyrhynchos*	D	highly pathogenic	CAZ, MEM, IPM, ETP, AMK
KPD 967-BA	FS30432	*Corvus monedula*	D	highly pathogenic	TZP, FEP, MEM, IPM, ETP, CN, NET, CIP, SXT, TGC

KPD 973-Ba	SN26343	*Anas platyrhynchos*	D	highly pathogenic	MEM, IPM, ETP, AMK, CN, NET, CIP, TGC
KPD 950 BA	FS17244	*Larus canus*	D	highly pathogenic	MEM, IPM, ETP, AMK, CN, NET, CIP

TZP – piperacillin / tazobactam, FEP- cefepime, CAZ - ceftazidime, IPM-imipenem, ETP- ertapenem, MEM - meropenem, AMK-amikacin, CN-gentamicin, NET-netilmicin, CIP -ciprofloxacin, SXT-co-trimoksazol, TGC-tigecycline
